# Factors associated with dental and medical care attendance in UK resident Yemeni khat chewers: a cross sectional study

**DOI:** 10.1186/1471-2458-12-486

**Published:** 2012-06-27

**Authors:** Saba Kassim, Ray Croucher

**Affiliations:** 1 Queen Mary University of London, Barts and The London School of Medicine and Dentistry, Institute of Dentistry, 4 Newark Street, London E1 2 AT, UK

## Abstract

**Background:**

The chewing of khat leaf with tobacco smoking amongst Yemenis, Somalis and Ethiopians is reported to impact oral and general health. The health status and particularly dental and medical care attendance of UK-khat chewers has not received attention. This study aimed to explore health status and dental and medical attendance and its associated factors in UK permanently resident Yemeni khat chewers.

**Methods:**

A cross- sectional study with a purposively selected sample of 204 khat chewers was conducted. Data were collected through face to face interviews. Data analysis included descriptive, Chi-square tests and binary logistic regressions.

**Results:**

The mean age for this study sample was 44.84 (SD = ±19.70) years and 35% were employed. Forty five percent reported symptomatic medical attendance for self reported health conditions in the last two weeks whilst 44% had symptomatic dental attendance. Higher khat chewing dependency levels associated positively with both symptomatic dental and medical attendance (p = 0.004, OR = 1.14, 95%CI = 1.04-1.25; p = 0.003; OR = 1.16, 95%CI = 1.05-1.29, respectively). Higher social participation levels associated negatively with symptomatic dental attendance (p = 0.034, OR = 0.98, 95%CI = 0.96-0.99) whilst increase in age and self-reported health conditions associated positively with symptomatic medical attendance (p = 0.030, OR = 1.03, 95%CI = 1.01-1.06; p = 0.001, OR = 4.51, 95%CI = 2.02-10.08, respectively).

**Conclusions:**

In this study of khat chewers, a significant proportion reported symptomatic dental and medical attendance. Demographic, psychosocial and self reported general health conditions were associated significantly with dental and medical attendance. Strategies to improve the dental and medical care attendance amongst this group should focus on these and other unexplored underlying factors.

## Background

The UK Yemeni community is one of the most established and yet least known of all its migrant groups [1]. Khat chewing, ‘a natural amphetamine’ [2], is used amongst this group facilitating social interaction in both homeland and diaspora [3]_._ Frequent khat chewing is associated with a range of oral and systemic health impacts [4,5] making it a national and international public health concern [6,7]. Yemeni khat chewers are also reported to have a high percentage of unemployment with low level of education, living in deprived areas and with low level of social participation [3,8]. High consumption of tobacco smoking and khat chewing dependency has been recently reported amongst this minority [3,8].

The health status and dental and medical care attendance amongst the UK’s Black and Minorities Ethnic Groups (BMEG) and globally have been studied extensively [9]. In the UK BMEG have generally poor health compared with the overall population [10]. Different factors are reported to associate with dental and medical attendance amongst minorities and drug users compared to the general population [11-14].

Currently, there is a gap in the literature with respect to dental and medical attendance in UK resident Yemeni male khat chewers. Though the evidence for dental and medical attendance in preventing oral problems and promoting better health outcomes is equivocal and complex [15-18], regular dental and medical attendance in this community is desirable because of its multiple disadvantages (low socioeconomic status, health risk behaviours such as tobacco and khat use).

**Figure 1:**
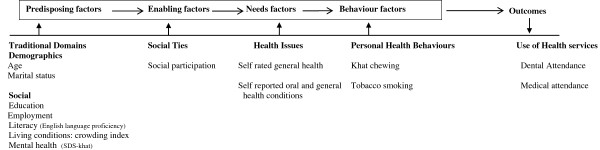
**Dental and medical care attendance for Yemeni khat chewers, drawn on Gelberg–Andersen Behavioral Model **[19]**.**

Exploring health service use (medical and dental attendance) amongst this group is important to create public oral and general health strategies. To facilitate our understanding of dental and medical attendance, an integrating conceptual framework (Figure 1) [19] was adapted. This model is commonly used to investigate health service use amongst minorities and substance abusers. This model also provides a conceptual framework that integrates predisposing characteristics (e.g. age, living conditions, ethnicity, literacy, and education), enabling factors (income, residence and social support) and need factors (professional and patient determined needs) that influence health care service use.

The aim of this study was to explore the relationship between Yemeni khat chewers’ characteristics that included socio-demographic, socio-economic, psychosocial and clinical factors (self-reported oral and general health conditions) with their dental and medical attendance. The study objectives were as follows: (1) to collect data describing adult Yemeni khat chewers’ characteristics (2) to describe the general and oral health and the use of dental and medical care service amongst this group and (3) to assess khat chewers’ associated factors with self-reported dental and medical attendance. It was hypothesized that like other substance users and BMEG, there would be similar symptomatic health care services use amongst resident Yemeni male khat chewers.

## Methods

### Sample size estimation and selection

This cross sectional study’s methods and sampling are described in Kassim et al. [20]. A purposively selected sample of permanently resident 204 male khat chewers age 18 years and older, speaking Arabic or English, permanent residents in the UK were recruited from khat selling outlets in Sheffield during random visits.

### Measures

Information was collected from khat chewers through a structured face to face interview [21] which was composed of six sections that assimilated items proposed in the conceptual framework (Figure 1).

### Predisposing factors

The predisposing characteristics (Figure 1) investigated here were age and marital status (married or other), employment status (employed or unemployed), status of education completed (high or low), language preferred for reading (English or both Arabic and English or Arabic and other languages), living conditions (uncrowded, crowded housing). Khat dependency was assessed using SDS-Khat [20]. This scale assessed chewers’ khat chewing dependency. It is composed of 5 items (range 0–15) with ≤ 5 or ≥ 6 scores meaning less or more likely dependence on khat.

### Enabling factors

The social participation of respondents was used as an enabling factor and was assessed using an index of social participation [21].

### Self-reported health needs factors

Self reported needs for both oral and general health conditions were investigated, using a dichotomised health related question ‘Do you have any health condition(s)?’. Respondents answering positively were asked to report the health conditions they had and their responses were summed [21]. Additionally, general health status was investigated using the validated item of ‘How would you describe your current health status?’ and responses were ‘Very good’, ‘Good’, ‘Fair’, ‘Bad and Very bad’. The former two categories were collapsed together and labelled as ‘uncompromised health’, and the latter three as ‘compromised health’.

Self-reported oral health status was also explored. A dichotomised oral health related question ‘Do you have any oral problem(s) such as pain /gum disease or any other?’ was asked. Those positively responding were asked to report all the oral problems they had and their responses were summed.

### Behaviour factors

Substance use included a composite 10 item khat behavioural measure [21] with ≥ 6 scores accounted as a high composite khat chewing behaviour. Tobacco use was investigated as ‘regular’ (at all times) or ‘episodic’ (only when chewing khat).

### Outcome measures

The dependent outcomes were symptomatic dental and medical attendance. Dental attendance was defined by the frequency of dental visiting and reason for dental visiting [22,23]. Reason for dental attendance was investigated and response categories were ‘Regular’, ‘Occasional’ and ‘Symptomatic’ attendance or ‘Never’ attended. Dental attendance was collapsed into two categories ‘Regular’ or ‘Occasional’ as ‘non symptomatic’ and ‘symptomatic’ attendance. The ‘never attended’ group were only included in the descriptive analysis as their attendance was not clear. For medical care use (GP contacts or visits because of health conditions in last two weeks) responses were coded as ‘non symptomatic’ and ‘symptomatic’ attendance.

### Ethical approval and confidentiality

This study was approved by The East London and City Health Authority Local Research Committee (No 05/Q06034/194). The confidentiality of the information obtained was assured and participant written consent was obtained.

### Data analysis

Data were analysed using SPSS v18. Descriptive analysis was run to report sample characteristics and to check that the assumptions of statistics tests were met. Frequencies were reported for categorical variables and mean and standard deviation for continuous variables if the assumption of normality was met. Otherwise the median is reported. Bivariate Chi-square (×^2^) tests were performed to identify any statistically significantly association at P ≤ 0.05 between explanatory independent and outcome variables. At this stage crude odd ratios and corresponding confidence intervals were also calculated. An independent samples T-test was performed, after checking the normality of the distributions, to compare the mean scores for social participation with the outcome variables. The Mann Whitney test was employed to compare the median scores of khat chewing dependency (SDS-Khat) and age in years for ‘symptomatic’ and ‘non symptomatic’ dental and medical attendance. Logistic regressions were employed and variables with significant associations (P ≤ 0.05) with health outcomes were entered into a multivariate model, based on the proposed theoretical background (Figure 1) [19]. Multicollinearity was checked between independent variables. SDS-khat instead of the composite khat chewing behavior score, age instead of employment status and self reported general health conditions instead of self rated health were entered into the model.

## Results

### Sample general characteristics, tobacco smoking and khat chewing behaviours

The sample’s mean age was 44.84 (SD = ±19.70) (range = 18-87) years, 77% were married, 35% were employed and 66% had low level of completed education. The mean score for social participation was 41.24 (SD = ±19.55) (median = 39) (range 2–102). The mean score for the SDS-khat was 5.52 (SD = ±4.03) and the median was 6.00 (range 0.00-15.00). Of the respondents 49% were less likely khat dependent (≤ 5.00 scores). The estimate of tobacco smoking regularly or when chewing khat was 65% and 36% of respondents reported high composite khat chewing behaviours.

### Aspects of oral and general health and health care attendance

Twenty nine percent of respondents reported oral problems that included dental decay or missing teeth (77%), gum problems (47%) (inflammation, pockets and gum pain) or other (15%) (13% dry mouth and 2% Temporo-mandibular joint problems). The mean number of self-reported oral problems was 1.61 (SD = ±0.49) (median = 2, range 1–2). Aspects of dental attendance and self-reported oral health conditions are shown in Table 1.

**Table 1:** Health aspects of study sample (n = 204)

**Variables**	**F (%)**
**Dental attendance**	
Regular & Occasional dental attendance	78 (38.2)
Symptomatic attendance	90 (44.1)
Never attend	36 (17.6)
**Self-reported oral health condition(s)**	
No	144 (70.6)
Yes (One oral health condition or more)	60 (29.4)
**GP contact or attendance**	
No	113 (55.4)
Yes	91 (44.6)
**Self-reported health condition(s)**	
No	127 (62.3)
Yes (One health condition or more)	77 (37.7)
**Self-rated health status**	
Uncompromised health	122 (59.8)
Compromised health	82 (40.2)

Thirty eight percent of respondents reported health conditions (Table 1). The mean number for health conditions among the 77 reporting health conditions was 1.61 (SD = ±0.92) (median 1, range 1–5). Fifty eight percent reported one health condition and the remainder two or more. Health conditions reported were: cardiovascular diseases (30%), diabetes (29%), psychological health problems (17%) and arthritis (23%). Medical attendance in the last two weeks and self-rated health status of study participants are reported in Table 1.

### Bivariate and logistic regression results of factors associated with dental attendance

The mean score for social participation (47.69, SD = ±20.17) for ‘non symptomatic’ dental attenders was significantly higher than that for ‘symptomatic’ attenders (38.71, SD = ±17.66). There were significant differences in the median scores for self-reported khat chewing dependency for ‘non symptomatic’ (Md = 4.00) and those with ‘symptomatic’ dental attendance (Md = 7.00). Age had no impact on dental attendance. The median scores for age for both ‘non symptomatic’ and ‘symptomatic’ dental attendance were alike (Md = 42.00 years old).

Table 2 shows factors associated significantly with ‘symptomatic’ dental attendance. Other factors that included marital status, English language proficiency, living conditions, employment status and tobacco smoking were not significantly associated with reasons for dental attendance.

**Table 2:** Frequency distribution and results of bivariate Chi-square tests of factors associated significantly with dental and medical attendance in a sample of UK resident adult male Yemeni khat chewers (n = 168)

**Explanatory factors**	**Dental attendance**						**Medical attendance**				
	**Non symptomatic F (%)**		**Symptomatic F (%)**		**Unadjusted OR (95%CI)**		**Non symptomatic F (%)**		**Symptomatic F (%)**		**Unadjusted OR (95%CI)**
**Marital status**											
Other status							29	(76.3)	9	(23.7)	1
Married							61	(46.9)	69	(53.1)	3.65 (1.60, 8.30)***
**Education level**											
High education	33	(58.9)	23	(41.1)	1		36	(64.3)	20	(35.7)	1
Low education	45	(40.2)	67	(59.8)	2.14 (1.11,4.10)*		54	(48.2)	58	(51.8)	1.93 (0.99, 3.74)*
**Employment status**											
Employed							51	(86.4)	8	(13.6)	1
Unemployed							39	(35.8)	70	(64.2)	11.44(4.93, 26.56)***
**Reading language**											
English, English & Arabic							43	(65.2)	23	(34.8)	1
Arabic & other							47	(46.1)	55	(53.9)	2.19 (1.16, 4.14)*
**Crowding**											
Overcrowded††							43	(42.6)	58	(57.4)	1
Uncrowded†							46	(69.7)	20	(30.3)	0.33 (0.17, 0.62)***
**Self-rated health**											
Uncompromised							73	(74.5)	25	(25.5)	1
Compromised							17	(24.3)	53	(75.5)	9.10 (4.47, 18.53)***
**Self-reported health condition(s)**											
No							76	(75.2)	25	(24.8)	1
Yes							14	(20.9)	53	(79.1)	11.51(5.48,24.18)***
**Self-reported oral health condition(s)**											
No	60	(52.6)	54	(47.4)	1						
Yes	18	(33.3)	36	(66.7)	2.22 (1.13,4.36)*						
**Composite of khat behaviour**											
Low	59	(53.6)	51	(46.4)	1		71	(64.5)	39	(35.5)	1
High	19	(32.8)	39	(67.2)	2.38 (1.22,4.61)*		19	(32.8)	39	(67.2)	3.73 (1.91, 7.33)***

*p≤0.05, **p≤0.01, ***p≤0.001; 95%CI: 95%confidence interval, OR: Odd ratio;

†One person a room; ††More than one person in the room.

After controlling for age, increase in khat dependency levels was associated positively (p = 0.004, OR = 1.14, 95%CI = 1.04-1.25) with symptomatic dental attendance, whilst higher social participation was associated negatively with symptomatic dental attendance (p = 0.034, OR = 0.98, 95%CI = 0.96-0.99) (Table 3).

**Table 3:** Results of the hierarchical multivariate binary multiple regression predicting dental attendance in a sample of UK resident adult male Yemeni khat chewers (n = 168)

**Explanatory factors**	**Model 1**		**Model 2**		**Model 3**	
	**Predisposing factors**		**Predisposing and enabling factors**		**Predisposing, enabling and self-reported needs factors**	
	**B**	**OR ( 95%CI)**	**B**	**OR ( 95%CI)**	**B**	**OR ( 95%CI)**
**Age**	−0.008	0.99 (.97, 1.01)	−0.014	0.99 (0.97,1.01)	−0.016	0.98 (0.96,1.00)
**Education level**						
High education	0.682	1	0.627	1	0.567	1
Low education		1.98 (0.95, 4.11)		1.87(0.89, 3.96)		1.76 (0.83, 0.77)
**SDS-khat**	0.141	1.15(1.05,1.26)***	0.132	1.14 (1.04, 1.25)***	0.133	1.14 (1.04, 1.25)***
**Social participation**			−0.022	0.98 (0.96, 0.99)*	−0.020	0.98 (0.96, 0.99)*
**Self-reported oral health condition(s)**						
No					0.653	1
Yes						1.92 (0.93, 3.99)

*p≤0.05, **p≤0.01, ***p≤0.001;

Multiple Regression Model 1: N=168, Cox & Snell R Square and Nagelkerke R Square 8.8-11.7 %;

Multiple Regression Model 2: N=168, Cox & Snell R Square and Nagelkerke R Square 11.7-15.7 %;

Multiple Regression Model 2: N=168, Cox & Snell R Square and Nagelkerke R Square 13.4-17.9 %.

### Bivariate and logistic regression results of factors associated with medical attendance

The social participation mean score (46.13, SD = ±20.83) for ‘non symptomatic’ medical attenders was significantly higher than that for ‘symptomatic’ attenders (39.13, SD = ±17.66). Increase in the self reported khat chewing dependency scores (Md = 8) was associated significantly with ‘symptomatic’ medical attendance, compared with that for non-dependents (Md = 3). Likewise increase in age was associated significantly with ‘symptomatic’ medical attendance, Md = 61 years for older and Md = 32 for younger khat chewers respectively. Table 2 reports factors associated significantly with ‘symptomatic’ medical attendance.

Tobacco smoking and other factors such as marital status and English language proficiency were not significantly associated with reporting ‘symptomatic’ medical attendance.

Logistic regression modeling showed an increase in khat chewing dependency levels (p = 0.003, OR = 1.16, 95%CI = 1.05-1.29), increase in age (p = 0.030, OR = 1.03, 95%CI = 1.01-1.06) and self-reported health conditions (p = 0.001, OR = 4.51, 95%CI = 2.02-10.08), were associated significantly with ‘symptomatic’ medical attendance (Table 4).

**Table 4:** Results of the hierarchical binary multiple regression predicting medical attendance in a sample of UK resident adult male Yemeni khat chewers (n = 168)

**Explanatory factors**		**Model 1**		**Model 2**		**Model 3**	
	**Predisposing factors**		**Predisposing and enabling factors**		**Predisposing, enabling and self-reported needs factors**		
	**B**	**OR ( 95%CI)**	**B**	**OR ( 95%CI)**	**B**	**OR (95%CI)**	
**Age**	0.041	1.04(1.02,1.07)***	0.041	1.04(1.02,1.07)***	0.030	1.03 (1.01,1.06)*
**Marital status**						
Other status	0.982	1	0.963	1	0.931	1
Married		2.67 (.96, 7.42)		2.62 (.93, 7.41)		2.54 (.85,7.58)
**Education level**						
High education	−0.104	1	−0.096	1	−0.072	1
Low education		0.90 ( 0.42,1.93)		0.91 ( 0.42, 1.95)		0.93 (0.415,2.09)
**Reading language**						
English, English & Arabic	−0.290	1	−0.287	1	−0.284	1
Arabic & other		0.75 ( 0.35, 1.61)		0.76 (0.35,1.64)		0.75 ( 0.33, 1.70)
**Crowding**						
Overcrowded	−0.794	1	−0.798	1	−0.511	1
Uncrowded		0.45 ( 0.21, .98)*		0.45 (0.21, .98)*		0.60 ( 0.26,1.37)
**SDS-khat**	0.162	1.18 (1.07,1.29)**	0.164	1.18(1.03,1.39)***	0.152	1.16 (1.05,1.29)***
**Social participation**			0.002	1.00 ( .98,1.02)	0.008	1.01 (.99, 1.03)
**Self-reported health condition(s)**						
No					1.51	1
Yes						4.51 (2.02, 10.08)***

*p≤0.05, **p≤0.01, ***p≤0.001;

Multiple Regression Model 1: N=168, Cox & Snell R Square and Nagelkerke R Square 30.0-40.2 %;

Multiple Regression Model 2: N=168, Cox & Snell R Square and Nagelkerke R Square 30.0-40.2 %;

Multiple Regression Model 3: N=168, Cox & Snell R Square and Nagelkerke R Square 34.6.46.3 %.

## Discussion

This cross sectional study aimed to explore health status and dental and medical attendance and its associated factors in UK resident Yemeni khat chewers. The main findings of this study were (1) 44.1% and 44.6% of participants reported ‘symptomatic’ dental and medical care attendance, respectively; (2) higher social participation was associated negatively with symptomatic dental attendance whilst age and self reported health conditions was associated positively with symptomatic medical attendance; and (3) increased dependency on khat chewing was a common factor for both symptomatic dental and medical attendance.

The findings from this study have lent further support to the current literature. Health status and dental and medical attendance amongst this group is similar to other BMEG as well as drug users [9,24,25]. Older respondents reporting health conditions were more likely to have ‘symptomatic‘ medical attendance [26]. Self-reported oral health conditions did not predict a pattern of dental attendance which is in accord with other studies [12,27] . The use of dental services would be more likely explained by social structure, belief and enabling factors than need factors [13]. Additionally, though this study only considered the pattern of dental attendance and not the last visit our findings were consistent with the literature in that a significant proportion had never attended a dentist [28]. Furthermore, amongst this study sample social participation has been found to be protective from ‘symptomatic’ dental attendance. A review noted that, in general, social connectedness and participation is inversely related to risk-related health behaviours [29]. Social participation and networking have been reported as influencing favorable health outcomes and behaviours such as tobacco cessation and dental attendance [30,31]. It may be suggested that, social participation acts to enable khat chewers to regulate their khat chewing, exposing khat chewers to a protective network that may instill in them the value of non-symptomatic attendance.

This study provides data for the self-reported oral and general health status and dental and medical attendance for a unique sample of UK resident Yemeni khat chewers. In addition, whilst many studies report frequent khat chewing as a common risk factor for unfavorable oral and general health outcomes [4,5], the novelty of this study is that severity of dependency on khat chewing is the common factor in symptomatic dental and medical attendance. This finding adds khat chewing dependency as a specific factor influencing dental and medical attendance amongst khat chewers alongside other factors reported for BMEG and drug users [9,24,25].

### Clinical and research implications

Health professionals’ awareness of the role of khat chewing dependency as a common driver for dental and medical attendance should be addressed. The findings of this study may inform the co-ordination and exchange of experiences between health care professionals serving this community. Tailoring and integrating oral and general health promoting strategies should target this community as the literature reports a lack of community knowledge about khat chewing impacts [3,21,32]. Future research should explore, firstly, factors that may hinder medical and dental care service access and attendance [33] amongst this and other khat chewing communities of both genders, considering that UK medical care is currently publically funded. These factors and the protective effect of social participation could be addressed through the use of qualitative methods, driven by the proposed theoretical approach [19], involving key informants from this community [34], and finding a cross-cultural mechanism of communication. Secondly, further exploration of khat chewing and health, that may drive health service use, should be sought.

### Limitations of the study

The sampling frame of this study was khat sale outlets. Mapping outlets to establish a comprehensive sampling frame could have contributed to recruitment of a more diverse sample that may have allowed more understanding of both dental and the medical care attendance amongst this study sample [35]. This study recruited khat chewers and there is no knowledge of the dental and medical attendance amongst non-chewers from this community as well as for these who have private dental and medical care. The purposive sampling design adapted was aimed to comprehensively establish data for khat chewing and associated health related outcomes. Awareness, knowledge, attitude and health behaviours amongst this community as whole (khat chewers and non khat chewers) await future investigation taking into consideration that accessing the Yemeni community is currently acknowledged as difficult in the absence of a sampling framework (Sheffield Hallam University, unpublished observation). Self-reported dental and medical attendance was not verified with dental and medical records and as such this may weaken the validity of these study findings. Though the literature reports that many factors can affect validity of self reported data such as recall and willingness of participants to report it [36,37], the concordance of self-reported data and medical records is sufficient to allow the use of self-report [38,39]. Factors such as health beliefs and the health care system [24,26,40] which may have influenced dental and medical attendance amongst this group were not investigated. The cross sectional study design excluded any causality and generalizability of findings is not appropriate. The homogeneity of the sample in its deprivation status (high unemployment, low level of completed education, limited English language proficiency) meant that the importance of these factors in health services use was not supported [41-43]. This might be addressed through multicentre sampling.

## Conclusion

In this study of khat chewers, a significant proportion reported symptomatic dental and medical attendance. Demographic, psychosocial and self reported general health conditions were associated significantly with dental and medical attendance. Strategies to improve the dental and medical care attendance amongst this group should focus on these and other unexplored underlying factors.

## Competing interests

The authors declare that they have no competing interests.

## Authors’ contributions

RC was the principal investigator and proposed the study design, SK contributed to the study conception, review the literature, collecting and analyzing the data and drafting the article draft. Both authors read and approved the final manuscript.

## Pre-publication history

The pre-publication history for this paper can be accessed here:

http://www.biomedcentral.com/1471-2458/12/486/prepub
